# Pet Ownership, Pet Attachment, and Longitudinal Changes in Psychological Health—Evidence from the Baltimore Longitudinal Study of Aging

**DOI:** 10.3390/geriatrics10060156

**Published:** 2025-11-25

**Authors:** Erika Friedmann, Nancy R. Gee, Eleanor M. Simonsick, Barbara Resnick, Merve Gurlu, Ikmat Adesanya, Soyeon Shim

**Affiliations:** 1 Department of Organizational Systems and Adult Health, School of Nursing, University of Maryland, 655 W. Lombard St., Baltimore, MD 21201, USA; 2 Center for Human-Animal Interaction, Department of Psychiatry, School of Medicine, Virginia Commonwealth University, Richmond, VA 23298, USA; nancy.gee@health.vcu.org; 3 Baltimore Longitudinal Study of Aging, Intramural Research Program, National Institute on Aging, National Institutes of Health, Baltimore, MD 21224, USA; simonsickel@grc.nia.nih.gov; 4 School of Nursing, University of Maryland, Baltimore, MD 21201, USA; resnick@umaryand.edu; 5 Office of Research and Scholarship, School of Nursing, University of Maryland, Baltimore, MD 21201, USA; mgurlu@umaryland.edu (M.G.); soyeonshim@umaryland.edu (S.S.)

**Keywords:** human–animal interaction, quality of life, happiness, depression, anxiety, wellbeing, SF12, CES-D, attachment

## Abstract

Introduction: While pet ownership (PO) is generally associated with better psychological health, research does not consistently demonstrate this relationship among community living older adults. Pet attachment has been suggested as a mechanism for the health benefits associated with pet ownership. We examine the contributions of PO and pet attachment to maintaining psychological health among generally healthy, cognitively intact, community-dwelling older adults as they age. Methods: Older adults (N = 596; age: ≥50, M = 67.6, SD = 9.5 years, pet owners N = 178) completed PO history and assessments of anxiety, depression, happiness, and mental wellbeing every 1–4 years. Pet owners completed demographic and pet attachment assessments. Linear mixed models with random intercepts and covariates of initial age, sex, race, live alone, married, and comorbidities quantified longitudinal changes (M = 7.5, SD = 3.6 years) according to time-varying PO, pet attachment, and dog walking to these changes. Results: PO moderated changes in anxiety (*p* = 0.011) and happiness (*p* = 0.037), which improved in pet owners and deteriorated in non-owners, and in mental wellbeing (*p* = 0.007), which deteriorated faster in pet owners; PO was not related to changes in depression. Pet attachment was related to worsening mental wellbeing (*p* = 0.012). Dog walking was related to slower increases in anxiety (*p* = 0.005) and depression (*p* = 0.004). Conclusions: This study provides important longitudinal evidence that PO may reduce age-related decline in owners’ psychological health later in life. Pet attachment does not appear to be the mechanism for the advantages of PO. We suggest potential reasons. Additional research is needed to confirm mechanisms.

## 1. Introduction

With the aging of the population [1], there is an ever-increasing need for understanding behavioral mechanisms and the development of complementary and integrative interventions that may decrease time spent in functional decline with a corresponding decrease in Quality of Life (QOL) among older adults [2]. Maintaining psychological health is closely associated with maintaining overall health, as indicated by their integrative contributions via the biopsychosocial model [3].

### 1.1. Pet Ownership and Psychological Health

Pet ownership (PO) has been suggested as a lifestyle choice that might support the owners’ overall health and wellbeing [4]. Within the biopsychosocial model, pet ownership is conceptualized as a form of social support that directly impacts psychological health [5]. While previous research documented an association of PO with slower deterioration in both physical [6] and cognitive [7] function in USA older adults, little longitudinal research has explored the connection between PO and changes in psychological health with aging among older adults.

Cross-sectional evidence indicates that PO is associated with several aspects of psychological health, although there are studies with conflicting results. Most of these previous studies were conducted in convenience samples of adult populations that included older adults but not were not limited exclusively in older adults. We focus on older adults (individuals 50 years old or older) and the four indicators of psychological health: anxiety, depression, happiness, and mental wellbeing, that were assessed in the Baltimore Longitudinal Study of Aging (BLSA).

Within the context of the biopsychosocial model, pet ownership can be classified as a form of social support and directly impacts psychological aspects of health [8]. Previous studies have investigated the association of pet ownership with most of these indicators of psychological health in older adults.

While few studies address the association of PO with anxiety among older adults, the study designs are relatively strong based on Oxford Centre for Evidence-Based Medicine (OCEBM) criteria [9], and results generally associate PO with lower anxiety. One study from a population-based sample of older adults, which used propensity-matched analysis of 169 pet owners and 169 non-owners from the USA to control for biases related to who chooses to own pets, indicated an association of PO with lower anxiety [10]. Among 41 community-residing Australian older adults who owned pets and 27 non-owners that used an experience sampling approach and had the participants assess their moods six times per day for 7 days, there were no differences in anxiety between pet owners and non-owners [11]. However, the frequency of pet presence during assessments was associated with lower anxiety [11]. Among a large sample of 2551 Australian younger, older adults (age 60–64) anxiety symptoms did not differ between pet owners and non-owners [12]. An experimental study in the USA—in which half the adults of all ages with hypertension who wanted to own a pet were assigned to adopt and the other half were not—showed that obtaining a pet moderated the stress responses beyond the effects of anti-hypertensive medication [13].

A number of studies, limited to older adult participants, address the association of PO with depression. Depression either did not differ or was worse among pet owners than non-owners. Most of the cross-sectional studies, including the propensity-matched study mentioned above [10], a US national probability sample of 1232 older Americans [14], three studies using data from the Health and Retirement Survey, and another more recent nationally representative US sample of adults aged 50 and above did not find a relationship of PO with depression among older adults [15,16]. Two studies involving specific subpopulations of 88 older adults residing in a rural area who were homebound (unable to leave their homes on their own) and received meals delivered to their homes [17] and 1000 community dwelling older adults living in rural and urban areas of the southern US state of Alabama [4] also did not find relationships between PO and depression. In contrast, in two studies, PO was associated with depression. Depression was higher among 2551 Australian community-residing older adult pet owners (60–64 years) than non-pet owners when controlling for marital status and relationship status [12], and higher depression scores were related to higher odds of PO among a representative sample of 7617 British older adults (age > 50) after controlling for a number of covariates [18]. We did not find studies that indicated lower depression among older adult pet owners compared with non-owners.

Few studies address the relationship of PO with happiness, and the findings are inconsistent. In the experience sampling study mentioned above, PO was not associated with happiness, but the frequency of having pets present during assessments was related to happiness [11]. Other cross-sectional surveys that included validated measures of happiness did not find an association of happiness with PO. Two of the surveys included a few older adults, with ages of participants ranging from 19–68 from the USA and 19–63 years from Mexico and mean ages of 34 and 26 years, respectively [19,20]. In the first study, happiness tended to be greater among pet owners than non-owners; in the second, happiness did not differ between dog owners and non-owners. Of note, both were non-representative survey studies, and the latter was confined to residents of Monterey, Mexico. Results were similar among the USA older adults who provided data to the BLSA from March 2017 to March 2018 [21]. A few studies used less traditional methods to evaluate the relationship of PO to happiness and found evidence to support a relationship of PO with happiness. A study using information posted on social media as data found that overall pet owners, not limited to older adults, are happier than non-owners based on analytics related to facial expressions and valence estimates of the lexicon [22]. This method may be biased, since individuals are unlikely to post much about problems they are having with their pets. In a qualitative online survey of adults’ wellbeing, PO was mentioned by respondents in their definitions of happiness [23].

Few studies of the relationship of PO with mental wellbeing include only older adults, and the relationships they report are inconsistent. In a USA national representative sample of older adults, PO was associated with worse psychological wellbeing [16], and in an online survey of 498 older adults, PO was not associated with mental wellbeing [24]. Studies that included but were not limited to older adults reported consistent findings of a relationship of PO with better mental wellbeing. In an online survey of the general adult Malaysian population (age 18–61+), pet owners reported better mental wellbeing than non-owners during the COVID-19 Lockdown [25]. In a cross-sectional survey of the general Mexican population (N = 602), dog ownership was associated with better mental wellbeing [20]. Similarly, in a mixed-methods study among 167 Australian cancer survivors, PO was related to mental wellbeing [26]. In an experience sampling of adults 18+ years old, where pet presence and mood were assessed 10 times per day for 6 days, pet’s presence during assessments was associated with better mental wellbeing [27].

We are aware of only one study that examines an association of PO with changes in psychological health as older adults age. In a representative sample of 5334 British older adults (age > 50 years), depression did not change differently according to pet ownership from 2010/2011 to 2016/2017 after controlling for a number of demographic and health variables [18].

### 1.2. Pet Attachment and Psychological Health

Pet ownership is not always associated with psychological health. It is likely that any impact of PO will be associated with the owner’s relationship with their pet. Pet attachment, the strength of the emotional bond between a pet owner and their pet, is posited as a psychological support for pet owners that would positively impact the owner’s health [15,28,29,30]. In the context of the biopsychosocial model, pet attachment is conceptualized as making a direct impact on psychological health among pet owners. Pet attachment has been related to psychological health in several studies of older adults and as well as in studies of more age-diverse populations.

In an earlier, smaller sample of the American older adult participants in the current study, pet attachment was not significantly correlated with anxiety [21], but attachment was related to greater anxiety among Australian adults, including older adults [31]. Greater pet attachment was related to greater depression in a smaller group of community-residing (lived in their communities and not in assisted living or other care facilities) older adults who were early participants in the current study [21], rural older USA adults [32], and USA self-selected older adult women [28] and Australian adults, including older adults [31]. In contrast, in cross-sectional assessments of a self-selected group of USA older adult women, higher pet attachment was related to lower depression [33], while in other studies, pet attachment was not related to depression among USA cat owners [15] or in USA bereaved older adults [14]. Studies of pet attachment and mental wellbeing also produced varying associations. In the two studies that were limited to older adults, the former in the USA and the latter in Australia, pet attachment was not related to mental wellbeing [21,34]. The relationship of pet attachment to mental wellbeing in studies that included but were not limited to older adults produced mixed results. In a general community survey of New Zealand adult pet owners aged 21–79 years, pet attachment was related to better mental health/wellbeing [35], while pet attachment was related to psychological distress in a similar group of Australians with a somewhat wider (20–94 years) age range [31]. In an online survey of 137 adult pet owners of unstated ages, viewing pets as family members was associated with better emotional wellbeing [36].

### 1.3. Dog Walking and Psychological Health

Dog walking can be conceptualized within the biopsychosocial model as a biological or physical factor that can have direct impacts on psychological health. It can provide exercise and exposure to the outside world, both of which are associated with psychological health. This is supported by studies documenting that exercise reduced depression [37,38] and promoted mental wellbeing [39] of older adults. In the context of the biopsychosocial model, dog walking can directly impact the social component of health, and the social component of health directly impacts psychological health. In 1147 instances of a young woman walking past other unfamiliar pedestrians in Northern Ireland, the pedestrians initiated social interactions more frequently when she was walking with a dog than walking alone [40]. In a walk-along qualitative study of healthy adult dog owners (N = 10) in New Zealand, the dog owners reported social interactions with others and environmental interactions during dog walking as positive influences and anxiety about dog behavior and as negative influences on psychological wellbeing from dog walking [41].

### 1.4. Aims

Few studies examine the association of PO with changes in psychological health as older adults age. No studies of which we are aware evaluate the relationship of pet attachment or dog walking to changes in the psychological health of older adults as they age. To fill gaps in the literature, the current study was initiated to explore the association of PO to changes in psychological health among generally healthy community-dwelling older adults and to evaluate the relationship of pet attachment to pets and dog walking with these changes.

Our first objective is to examine the relationship of pet ownership to long-term changes in psychological health. Our second objective is to examine the relationship of pet attachment to long-term changes in psychological health. Our third objective is to examine the relationship of dog walking to long-term changes in psychological health. We hypothesize that pet ownership and dog walking are associated with slower deterioration in psychological health, and that pet attachment is associated with the magnitude of changes in psychological health.

## 2. Methods

### 2.1. Design

This study constitutes a longitudinal cohort study with the addition of retrospective PO data. It was conducted within the Baltimore Longitudinal Study of Aging (BLSA), a longitudinal study of generally healthy older adults, which provided longitudinal prospectively conducted psychological health assessments that were essential for this evaluation. The BLSA is conducted by the Intramural Research Program of the National Institute on Aging component of the US National Institutes of Health. It began recruitment in 1958, is the longest running study of its kind, and continues recruiting healthy adults. Measures of psychological health are included among the questions asked during regularly scheduled BLSA assessments, which occur every one to four years depending on the participant’s age. Older individuals are assessed more frequently; those over 80 are assessed every year, while those from 60 to 79 are assessed every 2 years, and those under 60 every 4 years. The participants in the current study included all BLSA participants who were 50 years of age or older, completed the human–animal interaction module during a visit to the BLSA between March 2017 and March 2020, and were not deemed cognitively impaired. Impaired cognitive status was identified by consensus case conferencing that included interviews with family members and clinical impressions. Individuals who were judged as potentially impaired were excluded from the current study, since their ratings on the tools used for assessment of psychological health outcome variables and attachment may not be valid.

The PO data that were merged with the BLSA’s psychological health data were obtained with the addition of an HAI questionnaire to the BLSA assessments beginning in March 2017. The BLSA study and the addition of the HAI questionnaire were approved by the National Institutes of Health Intramural Research Program Institutional Review Board (#03-AG-0325). All participants provided written informed consent prior to participation.

Pet ownership and psychological health assessment data cover up to 13 years for each participant. This includes data from the regularly scheduled BLSA assessments for the ten years prior to the first administration of the HAI questionnaire and the three years of continued BLSA assessments after this survey visit, to the end of the data collection period (March 2020) for this study. A schematic representation of the HAI assessments and retrospective PO history within the context of the BLSA is available elsewhere [6]. Briefly, data were collected at a survey visit that included a 10-year PO history (see below). The data from the PO history were matched with demographic and psychological health data collected as part of each BLSA periodic assessment. The first year that included data from both the PO history and the BLSA assessments was considered the index visit and served as the baseline for the longitudinal analyses.

### 2.2. Instruments

Pet ownership-related measures were obtained from the HAI questionnaire. They include a 10-year PO history questionnaire, (1) a question assessing dog walking from the NIA-funded Health and Retirement Study (HRS) (NCHS 2020), and (2) the Lexington Attachment to Pets Scale (LAPS).

The 10-year PO history questionnaire was designed for this study. It gathers information about species of pets owned in each of the past ten years.

The LAPS includes 23 items about the owner’s attitudes towards pets, each on a 4-point Likert scale, with negatively worded questions assigning reverse codes [42]. LAPS was validated in a representative population sample of older adults (∝ = 0.93). Higher scores indicate greater pet attachment.

Four indicators of psychological health were assessed with well-validated instruments that were included in BLSA assessments: anxiety, depression, happiness, and mental wellbeing.

Anxiety was assessed with a subset of six items from the Perceived Stress Scale (PSS) [43], a 14-item scale that measures the degrees to which life situations are perceived as stressful. The PSS has shown good reliability in predicting the impact of stressful life events on humans with a reliability (∝) of 0.84, 0.85, and 0.86 in three different sets of adult samples [43]. The questions used to address stress included inability to control important things in life, nervousness, confidence about handling personal problems, difficulty coping, things not going their way, feeling upset from unexpected events, and being on top of things. Cronbach’s alpha for the reduced scale in the current sample is 0.74. Higher PSS scores indicate higher perceived anxiety.

Depression was measured with the Center for Epidemiologic Studies Depression Scale (CES-D), consisting of 20 four-point Likert scale (0–3) questions with a total score range of 0–60 [44]. The negatively worded questions are reverse-coded. The CES-D has demonstrated validity and reliability in a representative sample of US adults aged 24 to 74 years with an internal consistency of ∝ = 0.90 [45]. Higher CES-D scores indicate more severe depressive symptoms.

Happiness was measured using a single-item questionnaire where the participants rated their happiness between 0 and 10. This measure was deemed appropriate to measure happiness with a temporal stability (r = 0.86) and good concurrent and convergent validity [46]. Higher scores indicate greater happiness.

Mental wellbeing was assessed with the SF-12 Mental Component Summary score (MCS) [47], a reliable measure of mental health quality of life. It was validated decades ago to measure mental health components in the general population [48]. The SF 12 MCS mostly assesses role functioning (emotional), mental health, and social functioning. The MCS was demonstrated as a reliable (∝ = 0.86) and valid (ICC = 0.59) measure of mental wellbeing in a US older adults [49]. The MCS also was validated by association with social functioning, emotional role, and mental health (r’s = 0.53–0.58) [50]. Higher scores indicate better mental wellbeing [51]. Descriptive analyses revealed skew <−1.0 during assessment of assumptions for linear mixed models. To achieve normality required for outcomes in the analyses, mental wellbeing was reflected and natural log transformed (MCSrln) [52]. Subsequent analysis revealed adequate bivariate and multivariate normality. Thus, in the results presented in this study, better mental wellbeing is indicated by a lower score on the outcome.

Comorbidity scores at each BLSA assessment were based on the presence of common health conditions associated with poorer physical function and mortality. The comorbidity score is the sum of the number of eight conditions [heart disease (including angina pectoris, myocardial infarction (MI), congestive heart failure (CHF), angioplasty, coronary artery bypass graft (CABG)), diabetes, pulmonary disease, cerebral vascular disease, lower extremity arthritis, lower extremity pain, minor functional difficulty, and exertional pain while walking] the participant affirmed experiencing [21].

### 2.3. Statistical Analyses

Descriptive statistics were used to examine the data, identify outliers, and evaluate assumptions. Comparisons of demographic and baseline functional characteristics between dog owners and cat owners used t or chi square tests and excluded individuals who reported owning both cats and dogs. Little’s test indicated that data were missing completely at random.

Unconditional intraclass correlations (ICCs) confirmed considerable dependence of all psychological health outcome measures (anxiety: ICC = 0.583; depression: ICC = 0.583; happiness: ICC = 0.223; mental wellbeing: ICC = 0.403). The relationships of age and comorbidities to the outcomes were examined. Having fewer comorbidities was associated with greater anxiety (b = 0.052, se = 0.005, *p* = 0.013) and depression (b = 0.024, se = 0.011, *p* = 0.027), controlling for age. Older age was associated with lower anxiety (b = −0.081, se = 0.013, *p* < 0.001) and poorer wellbeing (r = 0.052, se = 0.024, *p* = 0.032), controlling for comorbidities. Based on the relationships of age and comorbidities to the outcomes, the relationship of sex, race (black vs. non-black), marital status, and living alone with psychological health, and to provide consistency in analyses, age at index visit, sex, and race as well as living alone, being married, and comorbidities at each visit were used as covariates in all multivariable analyses.

A linear mixed model (LMM) approach with a random intercept for participants was used to examine changes in each psychological health outcome over up to 13 years and the relationship of PO to these changes. In all longitudinal analyses, race, sex, and age at index visit were included as constant covariates. PO, living alone, being married, and comorbidities were included as time-varying covariates; thus, their values at each assessment were used as predictors of the outcome at that visit. Covariate inclusion was based on superior AIC and BIC model fit indices. Time was coded as years after index visit. The relationship of pet attachment to changes in psychological health outcomes was examined with LMMs for the entire group of pet owners. A final set of LMMs was used to examine the contribution of the binary variable of dog walking to changes in the psychological health outcomes of dog owners. Graphs of estimates from LMMs were created to help visualize moderation.

### 2.4. Participants

Participants included 596 cognitively intact older adults aged 50 years or older, 178 of whom owned pets. Demographic and initial descriptors of the entire sample and pet owners and non-owners are included in Table 1. At the index assessment, 78 participants kept dogs and 65 kept cats; 9 kept small mammals and 2 kept fish; and none of the participants reported keeping reptiles, amphibians, or other pets. Pet owners were significantly younger, more likely to be white, married or partnered, live with others, and reside in single-family housing than non-owners. Pet owners and non-owners did not differ significantly in comorbidities or mini mental status scores. There also were no significant differences in the psychological health variable scores between pet owners and non-owners at the index visit.

## 3. Results

### 3.1. Changes in Psychological Health Outcomes with Aging

Some measures of psychological health demonstrated significant changes with aging. In unadjusted analysis, depression (b = 0.8741, se = 0.0160, *p* < 0.001) increased and anxiety decreased (b = −0.2784, se = 0.0105, s = 0.008), while happiness (b = −0.0551, se = 0.0213, *p* = 0.796) and mental wellbeing (b = −0.0009, se = 0.0012, *p* = 0.941) did not change. Controlling for age, sex, race, live alone, married, and comorbidities, depression symptoms increased (b = 0.066, se = 0.025, *p* = 0.007) and mental wellbeing worsened (b = 0.006, se = 0.002, *p* < 0.001) significantly with aging. Stress (b = −0.019, se = 0.016, *p* = 0.246) and happiness (b = −0.009, se = 0.027, *p* = 0.708) did not change significantly when considering the entire sample over the course of the study.

### 3.2. Moderation of Changes in Psychological Health by Pet Ownership

**Figure 1:**
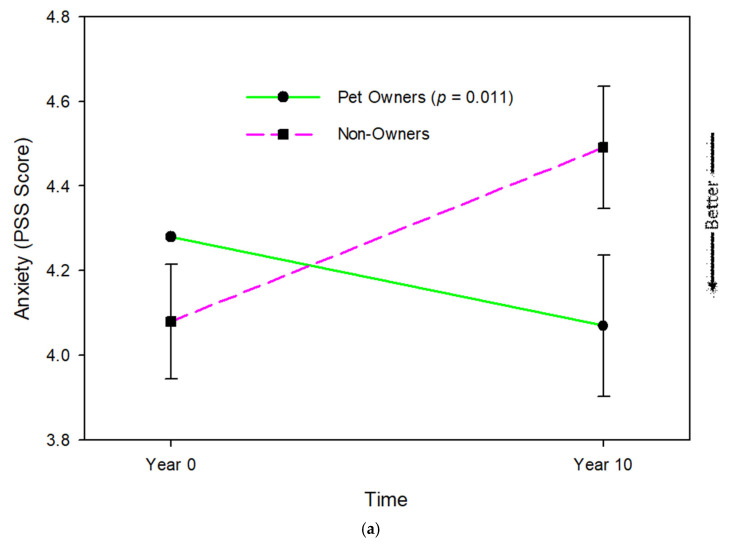

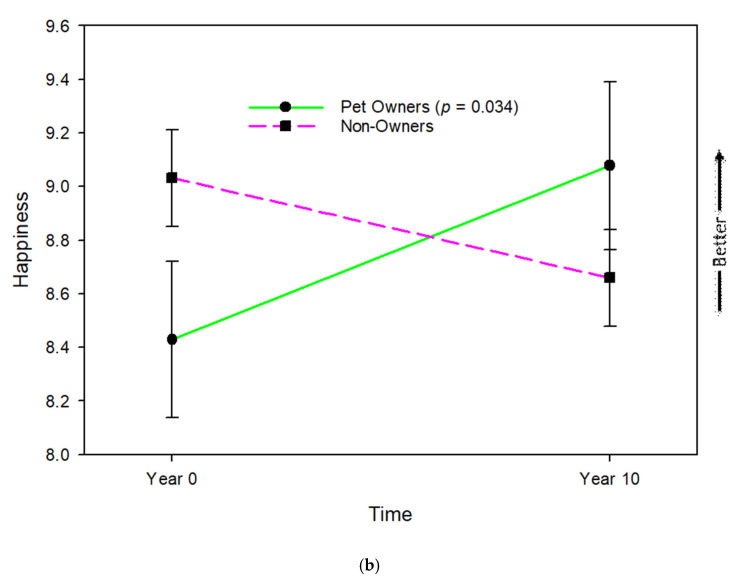

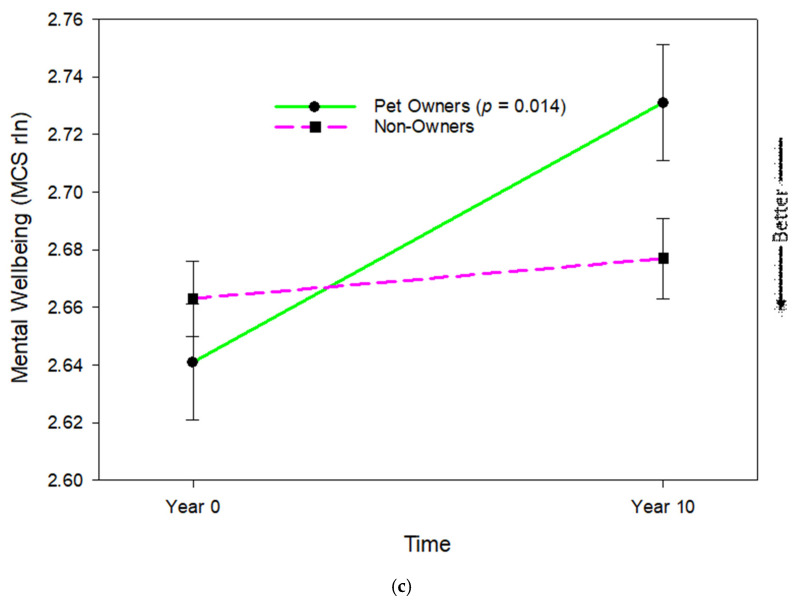
Changes in (**a**) anxiety, (**b**) happiness, and (**c**) mental wellbeing with aging in community-residing older adults (N = 596) according to pet onwership status from linear mixed models controlling for age, sex, race, lives alone, married, and comorbidities.

Pet owners and non-owners differed significantly in changes in psychological health with aging (Table 2). Pet ownership moderated changes in anxiety (*p* = 0.011), happiness (*p* = 0.037), and mental wellbeing (*p* = 0.007), but not in depression (*p* = 0.321). Anxiety decreased and happiness increased in pet owners, and the reverse was true of non-owners. Mental wellbeing deteriorated faster for pet owners than non-owners (see Figure 1).

### 3.3. Pet Attachment and Changes in Pet Owners’ Psychological Health with Aging

**Figure 2:**
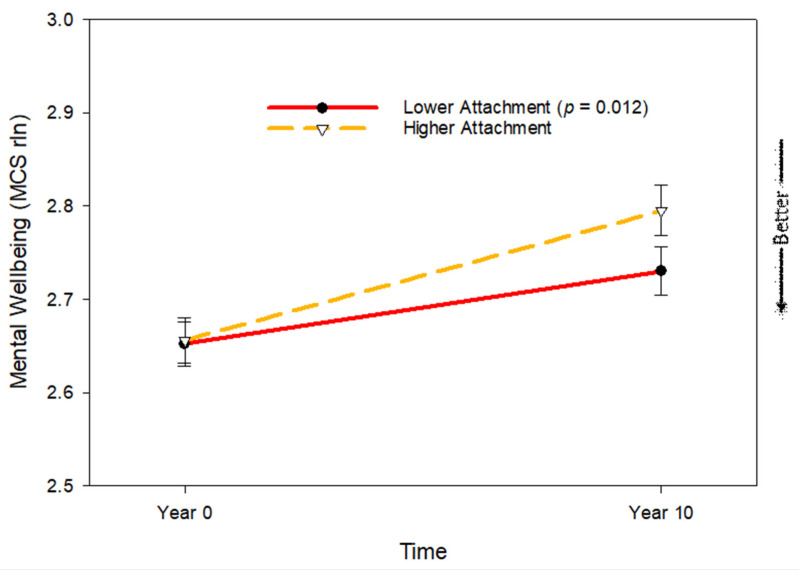
Changes in psychological wellbeing (SF-12 MCS reflected and natural log transformed) according to pet attachment. Estimates are at 1SD above and 1 SD below the mean of attachment.

Pet attachment was related to changes in psychological health with aging. Controlling for age, living alone, being married, and comorbidities among pet owners, pet attachment was related to changes in mental wellbeing with aging (*p* = 0.010). The greater the attachment to a pet, the greater the decrease in wellbeing; see Table 3, Figure 2.

### 3.4. Dog Walking and Changes in Dog Owners’ Psychological Health with Aging

**Figure 3:**
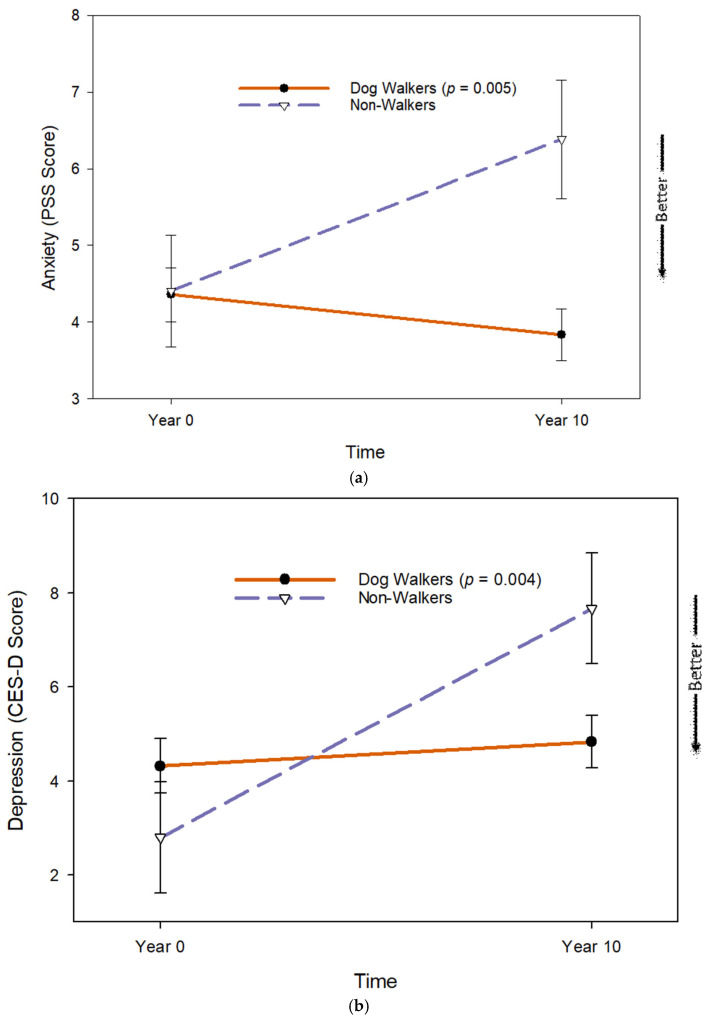
Changes in (**a**) anxiety and (**b**) depression according to dog walking.

Most dog owners (79.5%) reported that they walked their dogs. Changes in depression and anxiety among dog owners differed according to whether they did or did not walk their dogs [depression (b = 0.436, se = 0.149, *p* = 0.004); anxiety (b = 0.256, se = 0.090, *p* = 0.005)]. Dog owners who walked their dogs did not experience significant changes in anxiety or depression, while dog owners who did not walk their dogs experienced significant increases in both (Figure 3). Dog walking was not related to changes in happiness (*b* = −1.8569, se = 0.2451, *p* = 0.836) or mental wellbeing (b = 0.2861, se = 0.0093, *p* = 0.376).

## 4. Discussion

This study provides important longitudinal evidence that PO may promote some aspects of positive psychological health among generally healthy community-dwelling older adults by moderating age-related declines in psychological health later in life. Evidence did not support a relationship between pet attachment and changes in psychological health.

In the current study, depression increased with aging in both unadjusted and adjusted analyses, and the trajectory of change was not related to PO among the older adult participants. This finding is consistent with the lack of differences according to PO in trajectories of changes in depression found in a representative sample of older adults enrolled in the English Longitudinal Study of Aging [18]. One of the challenges with studying the relationship of PO to depression is that people may acquire or retain a pet to self-medicate for depression. In this case, we expect to see more depression among pet owners. Given this situation, it is important to research the association of depression to PO status among older adults, especially over time. Further research into reasons for PO and retention among older adults would help elucidate these differences.

In the current study, PO was related to improvements in anxiety and in happiness after controlling for age, sex, race, living alone, being married, and comorbidities. Anxiety did not intensify with aging overall, but anxiety decreased in pet owners and increased in pet non-owners. Findings with respect to PO and decreases in anxiety with aging complement the experimental study that indicated that adopting a pet lead to reduction in stress responses among younger adults [13] and an association of PO with lower anxiety among older adults [10].

While in the current study, PO was associated with improvements in happiness with aging, there are no longitudinal data for comparison. No prior studies addressed PO and changes in happiness with aging.

In the current study, PO was related to faster deterioration in mental wellbeing, controlling for age, sex, race, living alone, being married, and comorbidities. This is consistent with findings of a relationship of PO with better wellbeing in studies that include but are not limited to older adults [20,25,26] and a relationship of PO with similar [24] or worse [16] mental wellbeing in studies that are limited to older adults. The faster deterioration in mental wellbeing among pet owners contrasts with the ecological momentary association of mental wellbeing with the presence of pets during their owners’ daily lives [27]. It also contrasts with slower deterioration in physical wellbeing among pet owners [6]. The differences in the relationships of PO to changes in mental wellbeing with those of happiness, anxiety, and depression suggest that PO is associated with faster deterioration in emotional role functioning and social functioning with aging but slower deterioration in other aspects of mental wellbeing such as happiness, anxiety, and depression. It also is possible that some older adult pet owners may be more frustrated than non-owners with declines in their physical and cognitive function leading to poorer mental wellbeing.

It is possible that increasing physical and cognitive functional limitations with aging made it more difficult for pet owners to perform pet-related activities and thereby influenced their perceptions of their own wellbeing. Pet ownership can also reduce the owners’ participation in social activities, which promote psychological health. For example, among older adults almost 25% of pet owners, 30% of dog owners and 20% of cat owners reported that they declined to visit family or friends out of concern for their animals’ welfare [21].

The observed long-term deterioration in mental wellbeing among pet owners may also be related to pet loss or owner’s concerns about their ability to care for a pet. While the current study used PO at each BLSA assessment, it did not obtain data related to pet loss or pet acquisition. However, a recent systematic review revealed a clear correlation between high attachment and emotional distress following pet loss or caregiving burden [53], providing support for this potential explanation. We observed that individuals changed PO status over the course of this study, going from pet owners to non-owners and vice versa in approximately the same numbers.

The differing patterns of changes in psychological health between pet owners and non-owners that are reported in the current paper span years. These changes are occurring in the context of many other influences on participants’ lives. This contrasts with short-term changes that are observed from before to after animal-assisted interventions. Furthermore, the changes observed in this study occur with pet ownership, which puts very different burdens on pet owners compared to the situation where a dog or cat owned and handled by its owner visits for a short time.

This study provided no evidence for a relationship between pet attachment and improvement in psychological health with aging. In the current study, higher attachment was related to decreases in mental wellbeing among pet owners. Pet attachment was not related to changes in other psychological health outcomes for pet owners. This contrasts with the relationship of pet attachment to slower deterioration in physical function and executive function with aging among pet owners. Generally speaking, the role of pet attachment to psychological health or illness for older adults is not entirely clear; however, it does appear that high attachment is inversely related to wellbeing. This may suggest that older adults may experience an over-reliance on their pets for social support. If this conjecture is accurate, it would suggest that older adults who are highly attached to their pets may experience social limitations related to the need to be with their pets. For example, they may choose not to travel, or they may limit their travel to pet-friendly forms of transportation, often requiring them to drive their own vehicles, which may add an additional burden. It is also possible that the inverse relationship between high attachment and wellbeing may be connected to pet-related caregiving stress, anticipatory grief, or emotional dependence on their pet. All of these possibilities merit further elucidation through future research and are beyond the scope of this study.

The findings taken together raise questions about why and when older adults continue to keep pets. Pet attachment may play a role in older adults choosing to keep pets as they lose their memory and their psychological wellbeing deteriorates. Likewise, concern for a pet’s wellbeing and their own ability to care for that pet may provide a reason for older adults not to acquire a new pet following the loss of a previous pet. Furthermore, this relationship could differ depending on the species and even the characteristics of their pets.

Dog walking was associated with improvement or no changes in anxiety and depression, while anxiety and depression increased among dog owners who did not walk their dogs. This is consistent with a recent meta-analysis confirming the widespread belief that walking is an effective intervention to reduce depression symptoms [54]. Research supports that dog walking encourages social interactions [40] and contributes to neighborhood social capital [55], which affects pet owners and non-owners alike [56]. Assuming that dog walking is beneficial for older adults, public policy should provide support for older adults to maintain their pets, even if they are displaced into care facilities. The potential benefits to their owners’ psychological health warrant a serious exploration of this issue.

Examining each of the outcomes separately underscores that they may be assessing different aspects of psychological health. The findings with respect to mental wellbeing are consistent; for other aspects, they are not. Mental wellbeing deteriorated with aging, deteriorated more quickly for pet owners, and greater deterioration was related to greater pet attachment. The lack of an association of pet attachment with slower deterioration in psychological health is also consistent with previous findings with respect to changes in memory but inconsistent with findings with respect to other aspects of cognitive and physical function in this population [8]. While the current study addresses associations rather than causal relationships, the findings suggest that PO and pet attachment may play a more important role in maintaining health for some groups of older adults than others. This requires further elucidation.

Pet owners generally have responsibility for caring for their animals and expenses related to the animal’s care. Often, they must adjust their lives to accommodate the animals’ needs. They also experience pet illness and loss, and their psychological consequences [34,57,58,59]. These aspects of pet care warrant additional scrutiny in further studies of the longitudinal changes in health among pet owners. Additional longitudinal population studies are needed to support the causality of the changes in psychological health that were observed in relation to PO.

Differences in the trajectories of changes with aging in anxiety, depression, happiness and mental wellbeing between individuals who own pets suggest the importance of developing policies and strategies for maintaining the ability of older adults to keep pets and to have safe opportunities to walk their dogs as they age. These findings compliment previous evidence that pet ownership is related to slower deterioration in cognitive and physical function. We suggest an integrated analysis of the inter-relation of PO, with a focus on pet species, to the physical, cognitive, and psychological aspects of aging. We also hope that future research will rely on a more diverse and representative sample to examine not only the impact of PO but also animal interactions in a longitudinal manner to parse out the role of attachment to animals and the connections to psychological health or disease.

### Limitations

The largest limitation in the current study is the lack of diversity of the participants. While there was good representation of men, the participants were over 70% white, non-Hispanic, well educated, generally economically secure, and residing in predominantly in single-family homes. It is entirely possible that our sample is healthier, wealthier, and more socially active than is typical. This sample therefore lacks important diversity to be generalizable to the larger population of pet owners in the United States. This study was powered to examine changes in pet owners compared with non-owners. The smaller numbers of pet owners (n = 178) who were used to examine the relationship of attachment and dog walking (n = 78) to changes in psychological health outcomes reduced the power for those analyses.

The current study was designed as a longitudinal observational cohort study. Participants were individuals who chose to obtain a pet and those who did not. This is a limitation of almost all research addressing pet ownership. It is possible that the baseline characteristics that determine pet ownership also are those that determine the longitudinal changes that were observed. The covariates that are included in the current study limit but do not eliminate this possibility.

The use of data from the BLSA limited psychological health outcomes to those included in that study. Information about psychiatric comorbidities that were not assessed in the BLSA may have had important impacts on the findings. Additional measures, such as resilience, coping, or life satisfaction, would provide a more complete understanding of the relationship of PO and pet attachment to changes in older adults’ psychological health with aging. Furthermore, the moderating influences of some of these variables on the changes that were observed could also be warranted.

## 5. Conclusions

Among older adults, pet owners experienced less deterioration in anxiety and happiness and greater deterioration in mental wellbeing than non-owners. Pet attachment was only related to deterioration in mental wellbeing. The relationship of PO and pet attachment to changes in psychological outcomes with aging differs for different aspects of psychological health and between individuals. Findings compliment the previous findings that PO is related to slower deterioration in physical [6] and cognitive [7] function in this population of community-dwelling older adults.

This study provides important longitudinal evidence that PO may promote some aspects of psychological health among generally healthy community-dwelling older adults by moderating age-related decline in psychological health later in life.

## Figures and Tables

**Table 1:** Demographic and pet ownership characteristics at the BLSA index visit assessment, the first time for which PO and BLSA functional data both are available (elapsed time from baseline = 0) for study participants and according to pet ownership at index visit.

	All (N = 596)		Non-Pet Owner (n = 418)		Pet Owner (n = 178)		
Characteristic	N or M	(%) or (SD)	N or M	(%) or (SD)	N or M	(%) or (SD)	*p* ^†^
**Dog owner, N (%)**	78	(13.1)	0	n/a	78	(43.8)	
**Cat owner, N (%)**	65	(10.9)	0	n/a	65	(36.5)	
**Age in years, M (SD)**	67.6	(9.5)	68.8	(9.6)	64.8	(8.7)	**<0.001**
**Race: Black or African American, N (%)**	166	(27.9)	138	(33.0)	28	(15.7)	**<0.001**
**Sex: Female, N (%)**	337	(56.5)	234	(56.0)	103	(57.9)	0.671
**BMI, M (SD)**	26.9	(4.5)	26.93	(4.5)	26.70	(4.6)	0.570
**Comorbidities, M (SD)**	0.9	(1.2)	0.98	(1.1)	0.80	(1.2)	0.079
**Education: < College Graduate, N (%)**	72	(2.1)	59	(14.2)	13	(7.3)	0.211
**Married or partnered, N (%)**	40 0	(67.1)	263	(62.9)	137	(77.0)	**<0.001**
**Lives alone, N (%)**	150	(25.2)	119	(28.5)	31	(17.4)	**0.005**
**Single-family housing, N (%)**	493	(82.7)	325	(77.8)	168	(94.4)	**<0.001**
**Family income exceeds USD 50K, N (%)**	482	(80.9)	329	(78.7)	159	(85.48)	0.127
**Anxiety (PSS), M (SD)**	4.5	(3.0)	4.3	(3.0)	4.8	(3.2)	0.087
**Depression (CES-D), M (SD)**	4.6	(4.4)	4.8	(4.4)	4.4	(4.5)	0.352
**Happiness, M (SD)**	8.9	(5.6)	9.1	(6.6)	8.4	(1.4)	0.163
**Mental Wellbeing (MCS), M (SD)**	55.6	(5.6)	55.5	(5.6)	55.8	(5.4)	0.574

**N (%) indicates that the information that follows about this variable is in the form Number of Participants (% of Participants). M (SD) indicates that the information that follows about this variable is in the form Mean value (Standard Deviation)**. MMSE = Mini Mental Status Exam, PSS = Perceived Stress Scale, CES-D = Center for Epidemiological Studies Depression Scale, MCS = SF-12 Mental Component Score. ^†^ tests of significance are comparisons between pet owners and non-owners; *t* tests are provided for continuous variables, where M (SD) is indicated, Chi squared values are provided for categorical variables. Number of participants may be reduced due to missing data. Bold type indicates statistical significance at *p* < 0.05.

**Table 2:** Estimates of parameters for interaction of pet ownership with 10 years of aging from linear mixed models examining changes in psychological health, adjusted for age, sex, race, lives alone, married, and comorbidities among cognitively intact older adults (N = 596).

Outcome	Unstandardized Beta	Standard Error of Beta	*p*
Anxiety	0.062	0.024	**0.011**
Depression	0.036	0.038	0.322
Happiness	−0.102	0.048	**0.037**
Mental Wellbeing ^†^	−0.008	0.003	**0.007**

Anxiety = Perceived Stress Score, Depression = Center for Epidemiological Studies Depression (CES-D) score, Happiness = 1–10, Mental Wellbeing = SF-12 Mental Component Score reflected and natural log transformed. ^†^ Lower MCS score indicates greater wellbeing. Bold font indicates significant difference, *p* < 0.05.

**Table 3:** Estimates of parameters for interaction of pet attachment with aging from linear mixed models examining the contributions of pet attachment to changes in psychological health, adjusted for age, sex, race, lives alone, married, and comorbidities among cognitively intact older adults who kept a pet (N = 178).

Outcome	Unstandardized Beta	Standard Error of Beta	*p*
Anxiety	−0.0002	−0.001	0.908
Depression	0.0001	0.002	0.952
Happiness	0.0019	0.002	0.338
Mental Wellbeing ^†^	0.0004	0.0001	**0.012**

Anxiety = Perceived Stress Score, Depression = Center for Epidemiological Studies Depression (CES-D) score, Happiness = 1–10, Mental Wellbeing = SF-12 Mental Component Score reflected and natural log transformed. ^†^ Lower MCS score indicates greater wellbeing. Bold font indicates significant difference, *p* < 0.05.

## Data Availability

The dataset used for this study will not be publicly available. The study is ongoing, and the data are property of the National Institutes on Aging, Baltimore Longitudinal Study of Aging. Data are available through an application process available at the study website (https://www.blsa.nih.gov/how-apply, accessed on 9 April 2024).
